# Family functioning and suicidal ideation in college students: a moderated mediation model of depression and acceptance

**DOI:** 10.3389/fpubh.2023.1137921

**Published:** 2023-06-30

**Authors:** Biao Peng, Ningning Hu, Li Guan, Chao Chen, Zhu Chen, Huiying Yu

**Affiliations:** ^1^School of Marxism, Guizhou Medical University, Guiyang, China; ^2^Nursing School of Xinjiang Medical University, Urumqi, China; ^3^Hainan Women and Children’s Medical Center, Haikou, China; ^4^Zunyi Medical University, Guiyang Hospital of Stomatology, Guiyang, China; ^5^Guizhou Health Development Research Center, Guiyang, China

**Keywords:** college students, family functioning, suicidal ideation, depression, acceptance, moderating mediation

## Abstract

**Objective:**

To explore the mediating role of depression in the relationship between family functioning and suicidal ideation (SI) in college students, and to explore whether acceptance (It is one of the core components of psychological flexibility) plays a moderating role in this mediating model.

**Methods:**

In a cross-sectional study, questionnaires were distributed to college students during November and December 2022. The sample of Chinese college students (*n* = 592, 43.07% male, 56.93% female, mean age 19.40 years, SD = 1.24 years) completed the Family Adaptability and Cohesion Evaluation Scale (FACES III), the Center for Epidemiological Depression Scale (CES-D), the Positive and Negative Suicide Ideation Inventory (PANSI), and the Acceptance and Action Questionnaire-Second Edition (AAQ II). SPSS 25.0 for Windows and PROCESS 2.15 macros were used for data analysis.

**Results:**

There was a significant negative correlation between family functioning and SI, and depression played a mediating role in this relationship. Acceptance moderated the indirect effects of depression and SI in college students. In college students with a lower acceptance level (i.e., higher experiential avoidance level), depression had more influence on SI, while the influence of depression on SI was less in college students with a higher acceptance level (i.e., lower experiential avoidance level). Family functioning indirectly influenced SI through the moderation of acceptance.

**Conclusion:**

Mental health educators in colleges and universities should pay more attention to identifying and relieving depression in college students, thereby dealing with suicide risk more effectively. At the same time, college students should be discouraged from excessive use of experiential avoidance strategies, and instead taught to master effective emotional regulation strategies such as mindfulness, distress tolerance, and radical acceptance skills to improve their acceptance level and alleviate the influence of depression on SI.

## Introduction

Suicide is an increasingly serious public health problem. According to the World Health Organization, approximately 703,000 people worldwide die from suicide each year (1), and suicide is the fourth leading cause of death in the 15–29 age group (2). Studies show that, at 4.25 to 6.5 incidences per 100,000 college students, suicide is the second leading cause of death among college students (3). Suicidal ideation (SI) has been shown to be an important psychological basis for college students’ suicidal behavior, as well as the most sensitive predictor of suicidal behavior (4–6). SI is defined as thinking cognitively or imagining ending one’s life (7). A recent meta-analysis showed that the detection rate of SI among Chinese college students was 10.7% (8). While any incidence of SI should be of concern, the current incidence rate is this high indicates the importance, significance, and urgency of to studying SI in college students.

Previous studies have shown that the presence of issues in family functioning, depression, hopelessness, impulsiveness, and most mental disorders can predict SI (9–11). According to Family System Theory, the better one’s family functioning, the fewer emotional and behavioral problems one will exhibit, for example depression and SI in adolescents (12, 13). The Three-Step Theory of Suicide suggests that pain and hopelessness (which are central symptoms of depression) are the causes of SI (14). Therefore, depression may play a mediating role between family functioning and SI. Many studies have investigated the mechanism of family function’s influence on SI (e.g., depression, hopelessness, impulsivity), with other factors as mediating or moderating variables (15–17), however few studies have considered both depression as a risk factor and acceptance as a protective factor. In recent years, acceptance as one of the core concepts of Acceptance and Commitment Therapy (ACT) has received increasing attention. It is the opposite of experiential avoidance avoidance and can be effective in alleviating mental health problems (18). Therefore, based on theories related to Family System Theory and ACT, the current study aims to explore the mediating role of depression between family function and SI, as well as the moderating role of acceptance in this mediating model, so as to provide targeted guidance for the prevention and intervention of college students’ suicide.

### Family functioning and SI

Family is not only a basic unit within society, but also an important space for the healthy growth of the individual. The influence of family on adolescents’ physical and mental development is realized through family functioning, which is a comprehensive variable in measuring the overall quality of family operation (12). Olson’s Circumplex Model of Marital and Family Systems divides family functions into three dimensions: family cohesion, flexibility (or adaptability), and communication (19, 20). Family cohesion refers to the closeness of the relationship between family members. Family adaptability refers to the ability of the family to cope with the pressures of external environment or developments within the marriage. Family communication refers to the communication among family members. The better the function of all three of these dimensions, the better the mental state of the family members. Conversely, when these dimensions function more poorly, family members will be more prone to various mental health problems (21) and increased risk of suicide (22).

The Theory of Self-Determination holds that individuals have the basic need of maintaining intimacy and connection with their significant others, and that both individual or situational factors that contribute to the satisfaction of this basic need can improve an individual’s level of mental health. However, interfering with or hindering the satisfaction of this need will cause damage to the individual’s level of mental health and lead to SI (23). According to the Interpersonal Theory of Suicide proposed by Joyner (24), thwarted belongingness (the feeling of not belonging to one’s own social group) and perceived burdensomeness (the feeling of being burdened by significant others) are the causes of SI, and both are generated by the frustration of basic interpersonal needs (25). According to the Family System Theory, poor family functions (low cohesion, low flexibility, low communication) cannot meet the basic interpersonal and emotional needs of individuals, and cannot provide individuals with a sense of family intimacy, effective communication, and family participation, which will lead to a frustrated sense of belonging and make individuals feel that they are a burden to their families (26, 27). On the contrary, good family functions (high cohesion, high flexibility and high communication) can meet the basic interpersonal and emotional needs of individuals, provide them with a sense of family intimacy and participation, and make them feel the meaning of life, so as to enhance the sense of belonging and eliminate the sense of burden (28). Therefore, lower family functioning is difficult to meet the basic interpersonal needs of individuals, and will affect individuals’ SI through thwarted belongingness and perceived burdensomeness.

Empirical studies have also shown that family function has an important impact on adolescent SI (11). Family flexibility is significantly negatively correlated with adolescent SI (29, 30), and family cohesion is significantly negatively correlated with SI (31). Lack of family cohesion and flexibility in particular are an important predictor of SI (32). Based on the above-mentioned literature, then, we proposed our first hypothesis.

*Hypothesis 1*: There is a significantly negative correlation between family functioning and SI.

### The mediating role of depression

SI is not only affected by family functioning, but also directly related to depression. Studies have shown that individual SI is significantly correlated with depression, and depressive symptoms are strong predictors of SI (33, 34). SI is affected by depression. According to the Three-Step Theory of Suicide, both pain and hopelessness are important factors to explain suicidal behavior (9, 14), and both are important manifestations of depression [In fact, despair, helplessness, and hopelessness, are also part of the psychological structure definition of demoralization. Demoralization is a constant impotence that predicts suicidal ideation, along with feelings of helplessness, hopelessness, meaninglessness, subjective incompetence, and diminished self-esteem (35, 36)]. Depression often causes individuals feel hopeless and useless, making it easier for them to end their lives in an attempt to escape their pain, causing them to be more tolerant and receptive to the idea of suicide (37). The higher one’s degree of depression, the more understanding and support the individual will show toward suicide (38, 39).

Meanwhile, Family System Theory holds that families have a direct and important influence on an individual’s psychology and behavior (40). Good family functioning, such as a high level of family cohesion, can provide love and support (41), a high level of family flexibility to help adolescents cope with adverse events (42), improved positive communication which can reduce family conflicts (43), and thereby effectively reduce adolescent problem behavior and promote the healthy development of adolescents (44). In contrast, poor family functioning, such as low family cohesion, low family adaptability, and low levels of communication, will reduce a child’s psychological resources and lead to various problematic behaviors including depression (45, 46). Empirical studies have also shown that family functioning has an important impact on adolescent depression (47, 48), that family cohesion is significantly negatively correlated with depression (49, 50), and that low family adaptability and poor family communication are significantly negatively correlated with depression (51). Family functioning not only directly predicts individual depression, but also influences one’s depression through self-esteem (52). Family dysfunction will lead to individual depression, and depression will further lead to SI, causing one to be more inclined to support, affirm, and accept suicide as an option. Therefore, depression may be the mediating variable between family functioning and suicidal attitude. Based on this, we proposed our Hypothesis 2.

*Hypothesis 2*: Depression mediates the relationship between family functioning and SI.

### The moderating role of acceptance

Depression can predict SI, but not all depressed college students have the same level of SI. One possible reason for this could be that college students have different levels of acceptance of various negative feelings brought about by depression. Acceptance is one of the six core healing processes of ACT (the other five are contacting the present moment, defusion, self-as-context, value, and committed action). Acceptance refers to actively and consciously accepting the personal events that have occurred in one’s history, rather than unnecessarily trying to change their frequency or form (53), for example, consciously feeling the pain of depression rather than fighting it. It is not giving in, enduring, admitting defeat, wallowing, but making room with an open mind for all kinds of pain, feelings, impulses, and emotions (54). The counterpoint to acceptance is experiential avoidance, which is one of the six core elements of psychological inflexibility wherein one attempts to escape or avoid personal events, which causes the individual further psychological harm (54) and can produce mental health problems such as depression (55) and SI (56).

ACT suggests that acceptance and mindfulness bring about continuous and effective interventions and intervention components, which can provide relief and opportunity to adjust and change one’s experience of human suffering by relieving pain, despair, and other negative feelings caused by depression and thus reducing SI (18).

According to the Experiential Avoidance Model of deliberate self-harm, individuals using experiential avoidance to face depression and other adverse emotional reactions are more prone to self-harm behavior (57), however using acceptance to face depression and other adverse emotional reactions can alleviate self-harming behaviors.

Previous studies have shown that acceptance can effectively reduce one’s level of depression and SI (58), and a meta-analysis has shown that ACT can reduce SI by improving one’s level of acceptance and thereby improving their psychological flexibility (59). In addition, a recent study found that self-acceptance plays a moderating role between depression and SI (60). But self-acceptance is conceptually different from acceptance in ACT, and refers to the ability to think positively about yourself and your life, while acknowledging the good and bad qualities within yourself (60). Therefore, the moderating effect of acceptance on depression and SI is still worth studying. In conclusion, a high level of acceptance (i.e., a low level of experiential avoidance) may alleviate the influence of depression on SI in college students, while a low level of acceptance (i.e., a high level of experiential avoidance) may increase the influence of depression on SI in college students, and acceptance may play a moderating role in the relationship between depression and SI in college students. Based on the above research results, we proposed our Hypothesis 3.

*Hypothesis 3*: Acceptance plays a moderating role between depression and SI.

### The current study

**Figure 1:**
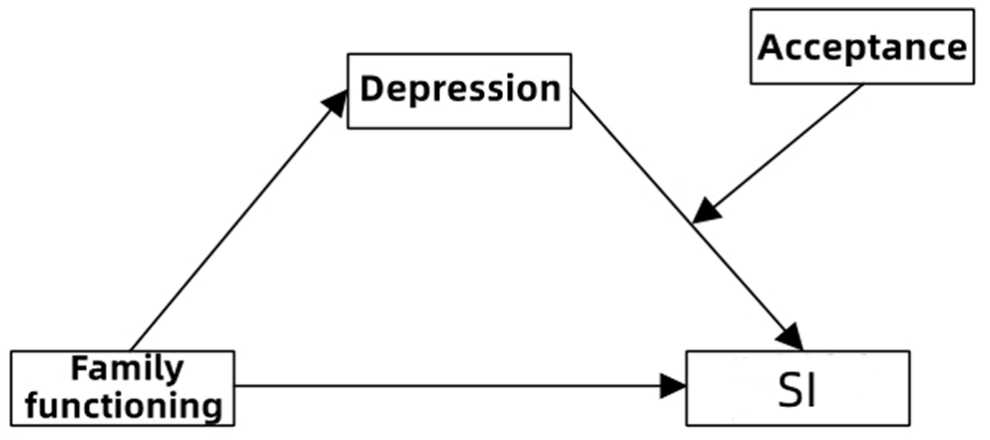
The proposed mediated moderation model.

Combining Family System Theory, the Interpersonal Theory of Suicide, and ACT, the present study aimed to explore a complex moderated mediation model underlying the association between family functioning and SI in college students (Figure 1). The purposes of our study were to (a) test whether family functioning significantly predicted SI; (b) test whether depression mediated the association between family functioning and SI; and (c) test whether acceptance moderated the association between depression and SI.

## Method

### Participants

A convenience sampling method was used to collect questionnaires from students attending a university in Guizhou Province, China. A total of 700 questionnaires were distributed, and 592 were collected after excluding invalid questionnaires (effective rate of 84.57%). The average age of respondents was 19.40 years (*SD* = 1.24; age: 18–24). The final sample comprised 255 boys (43.07%) and 448 girls (56.93%), and 240 freshmen (40.54%), 152 sophomores (25.68%), 116 juniors (19.59%), and 84 seniors (14.19%).

### Procedure

This study was approved by the Ethics Committee of Guizhou Medical University. The data was collected during November and December 2022. Prior to data collection, the participants were informed of the purposes of the study, and that all data collected would be anonymous and their responses would be kept confidential. All participants completed questionnaires in the classroom under the guidance of trained researchers.

## Measures

### Family functioning

The Family Adaptability and Cohesion Evaluation Scale (FACES III) developed by Olson (19) was used to measure family functioning. The scale is composed of 20 items measuring two dimensions: intimacy and adaptability. Intimacy refers to the emotional connection between family members, and adaptability refers to the adaptability of the family. Each item in the scale is scored on a five-point Likert scale which ranges from 1 (almost none) to 5 (almost always). The higher the total score of the scale, the better the family functioning. The Chinese version of the scale was revised by Xu et al. (61). In this study, the Cronbach’s α coefficient was 0.93.

### Depression

The Center for Epidemiological Studies Depression Scale (CES-D) as developed by Radloff (62) was used to measure respondents’ depression. The Chinese version of the scale was revised by Chen et al. (63). The scale consists of 20 questions, and responses are rated using a three-point scale ranging from 0 (occasionally or not at all) to 3 (most of the time or consistently) and consists of 20 questions. The Cronbach’s α coefficient of scale in this study was 0.92.

### SI

The Positive and Negative Suicide Ideation scale (PANSI) for Adolescents developed by Osman et al. (64) was used to assess respondents’ SI. The Chinese version of the scale was revised by Wang et al. (65), and uses a total of 14 questions to measure two dimensions, specifically, positive and negative SI. Positive SI is scored in reverse, and the total scores of positive and negative SI are added together to obtain a total score of SI. Responses are rated using a five-point Likert scale, ranging from 1 (never) to 5 (always). The higher the total score, the higher one’s level of SI. The Cronbach’s α coefficient of the scale in this study was 0.88.

### Acceptance

The Chinese version of the Acceptance and Action Questionnaire-Second Edition (AAQ II) was used to measure respondents’ level of acceptance (66). The scale measures a single dimension using a total of seven items, rated on a seven-point Likert scale ranging from 1 (never) to 7 (always). The higher the total score of the measure, the higher one’s experiential avoidance (i.e., the lower their acceptance). In this study, the Cronbach’s α coefficient was 0.92.

### Data analyses

A common method biases test was performed using SPSS 25.0 for Windows. The correlations among family functioning, depression, SI, and acceptance were investigated using Pearson’s product difference correlation analysis. The mediating effect of depression on family functioning and SI was tested using SPSS-process 2.15 macro (Model 4). SPSS-process 2.15 macro (Model 14) was used to test the moderated mediation effect. A simple slope test was used to analyze the moderating effect of acceptance on depression and SI. In current study, SI of college students had no significant difference in gender and grade (*t* = 0.51, *p* > 0.05; *F* = 0.76, *p* > 0.05), therefore, it is not necessary to include gender or grade as control variables. All tests used 5,000 bootstrap resamples to determine the 95% confidence interval (CI). If the 95% CI did not include 0, it indicated that the mediating effect, moderated mediating effect, and moderating effect were significant.

## Results

### Common method biases test

Harman’s single factor test was used to ensure data reliability and accuracy. The results showed that a total of 10 factors had characteristic root values greater than 1, among which the interpretation rate of the first factor was 28.37%, which is less than 40% and therefore indicated that there was no common method bias in this study.

### Preliminary analysis

Table 1 shows the mean value, standard deviation (*SD*), and correlation of all study variables. Family functioning was negatively correlated with SI (*r* = −0.36, *p* < 0.01), depression (*r* = −0.41, *p* < 0.01), and experiential avoidance (*r* = −0.32, *p* < 0.01). Depression was positively correlated with SI (*r* = 0.65, *p* < 0.01), and significantly positively correlated with experiential avoidance (*r* = 0.68, *p* < 0.01). Experiential avoidance was positively correlated with SI (*r* = 0.50, *p* < 0.01).

**Table 1:** Descriptive statistics and correlations for all variables.

	FF	Depression	EA	SI
FF	1			
Depression	−0.41**	1		
EA	−0.32**	0.68**	1	
SI	−0.36**	0.65**	0.50**	1
*M*	72.54	20.10	22.02	24.50
*SD*	12.84	7.72	7.37	6.44

**p < 0.01. FF, Family functioning; EA, Experiential avoidance; SI, Suicidal ideation.

### Mediation effect of depression

To test Hypothesis 2, the process macro Model 4 was used to examine the mediating effect of depression on family functioning and SI. As shown in Table 2, family functioning significantly negatively predicted SI (*β* = −0.36, *p* < 0.001) and depression (*β* = −0.41, *p* < 0.001), while depression positively predicted SI (*β* = 0.61, *p* < 0.001). The direct effect of family functioning on SI was significant (*β* = −0.11, *p* < 0.001). The bias-corrected percentile bootstrap method showed that family functioning had a significant indirect effect on SI of college students through depression: *ab* = 0.25, *SE* = 0.03, 95% CI = [−0.30, −0.19]. The mediating effect accounted for 69.44% of the total effect. Therefore, the mediating effect of depression on family functioning and SI is supported in Hypothesis 2.

**Table 2:** Testing the mediation effect of family functioning on SI.

	Model 1 SI		Model 2 Depression		Model 3 SI	
	*β*	*t*	*β*	*t*	*β*	*t*
FF	−0.36	−9.35***	−0.41	−10.80***	−0.11	−3.32**
Depression					0.61	17.97***
*R* ^2^	0.13		0.17		0.44	
*F*	87.38***		116.65***		228.90***	

**p < 0.01, ***p < 0.001. FF, Family functioning; EA, Experiential avoidance; SI, Suicidal ideation.

### Moderated mediation

The process macro Model 14 was used to test the moderated mediation effect of acceptance on depression and SI, as proposed in Hypothesis 3. As shown in Table 3, depression and experiential avoidance had significant interaction effects on the SI of college students (*β* = 0.07, *p* < 0.01). The bias-corrected percentile bootstrap method showed that the 95% CI for the cross term between depression and experiential avoidance did not contain 0 (95% CI = [0.03, 0.12]). Experiential avoidance (i.e., the opposite of acceptance) had a moderating effect on the indirect effects of family functioning and SI of college students, and its moderating index was −0.23, *SE* = 0.03, 95% CI = [−0.29, −0.17]. When the level of experiential avoidance was low (i.e., one standard deviation below the mean, that is, the level of acceptance was high), depression had a significant mediating effect on family functioning and SI of college students: *ab* = −0.20, 95% CI = [−0.25, −0.14]. Meanwhile, when the level of experiential avoidance was high (i.e., one standard deviation above the mean, namely, the level of acceptance is low), depression had a significant mediating effect on family functioning and SI of college students: *ab* = −0.26, 95% CI = [−0.33, −0.19].

**Table 3:** Testing moderated mediation effect of acceptance on depression and SI.

	Model 1 Depression		Model 2 SI	
	*β*	*t*	*β*	*t*
FF	−0.41	−10.80***	−0.11	3.21**
Depression			0.56	12.91***
EA			0.08	1.89
Depression × EA			0.07	3.27**
*R* ^2^	0.17		0.45	
*F*	116.65***		121.06***	

**p < 0.01, ***p < 0.001. FF, Family functioning; EA, Experiential avoidance; SI, Suicidal ideation.

**Figure 2:**
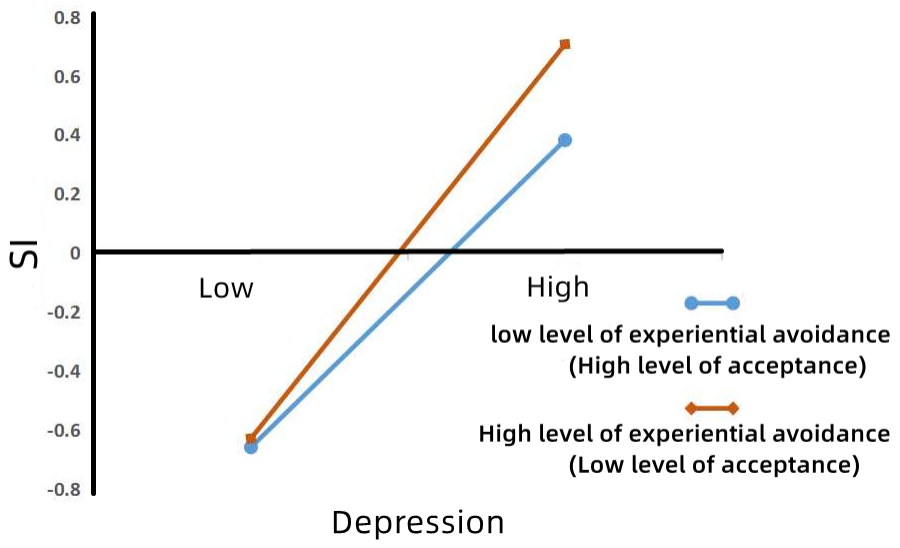
Model of test for simple slopes showing moderating influence of acceptance in the association between depression and SI.

To better understand the moderating effect of acceptance on depression and SI, a simple slope test was used (67). As shown in Figure 2, when the level of experiential avoidance was high (i.e., the level of acceptance was low), depression had a significant negative predictive effect on SI (*β*_simple_ = −0.67, *p* < 0.001). When the level of experiential avoidance was low (i.e., the level of acceptance was high), the negative predictive effect of depression on SI was decreased (*β*_simple_ = −0.52, *p* < 0.001). Therefore, a high level of acceptance was shown to alleviate the influence of depression on SI of college students, while a low level of acceptance was seen to increase the influence of depression on SI of college students, with acceptance playing a moderating role in the influence of depression on SI of college students.

## Discussion

Although numerous empirical studies have already explored the influence mechanism of family functioning on SI, few studies have considered the role of depression as a risk factor alongside acceptance as a protective factor in the influence of family functioning on SI of college students. Based on Family System Theory, Suicide Interpersonal Theory, and ACT, the current study explored the mediating role of depression and the moderating role of acceptance in the relationship between family functioning and SI of college students.

### Family functioning and SI

The results of the current study supported Hypothesis 1, that is, that the family functioning of college students is significantly negatively correlated with SI, which is consistent with the findings of previous studies (11, 32). According to the Interpersonal Theory of Suicide, SI stems from a sense of burdensomeness and thwarted belongingness (23). The burdensomeness is that individuals feel that their own incompetence causes stress to those around them (68). Meanwhile, thwarted belongingness is the negative psychological feeling generated when an individual’s basic needs are not met (69). According to Family System Theory, good family functioning (represented by cohesion, adaptability, and communication) can cultivate one’s positive self-esteem (52) and give one a sense of intimacy and belonging (70), and thus promote the individual’s mental health (12). However, poor family functioning may lead to one feeling a sense of impotence and burden, making it difficult for them to satisfy their need of a sense of intimacy and belonging, thus leading to SI (23, 32).

### The mediating role of depression

The results of the current study also supported Hypothesis 2, that depression plays a mediating role in the relationship between family functioning and SI in college students. This is consistent with findings of previous studies, that family functioning is an important predictor of depression (48, 52) and depression an important predictor of SI (34). The Circumplex Model of Marital and Family Systems divides family functions into family cohesion, flexibility (or adaptability), and communication (19). A high level of family cohesion can provide love and support (41), while a high level of family flexibility can help adolescents cope with adverse events (42), and active communication can reduce family conflicts (43), all of which both together and individually can effectively reduce depression and other mental health problems, and promote the mental health development of adolescents (44). In contrast, low family cohesion, low family adaptability, and a low level of communication will reduce adolescents’ psychological resources, leading them to depression, suicide, and other mental health problems (22, 45, 46).

According to the Three-Step Model of Suicide, a combination of pain and hopelessness leads to SI (14). Pain will then reduce their desire to live, thus triggering SI (71), and the despair triggered by the pain will strengthen SI (72). The interaction between pain and hopelessness is the fundamental reason for the generation and development of SI (14, 73). Pain and hopelessness are both key symptoms of depression (74). Individuals suffering from depression often feel hopeless, useless, and are prone to SI as they consider ending their lives to get rid of the pain (37).

To summarize, family function can predict SI and depression, depression can predict SI, and depression plays a mediating role between family function and SI in college students.

### The moderating role of acceptance

Our results also supported Hypothesis 3, that acceptance moderates the second stage of the indirect effect of family functioning on SI, that is, that acceptance moderates the relationship between depression and SI in college students. In college students with low level of acceptance (i.e., a high level of experiential avoidance), the correlation between depression and SI was stronger. However, this relationship was weakened among college students with high levels of acceptance and low levels of experiential avoidance. In other words, a high level of acceptance can mitigate the effect of depression on SI among college students.

This phenomenon can be explained by ACT. ACT proposes that experiential avoidance is a common emotion regulation function evident in a variety of strategies (75), and excessive use of experiential avoidance will lead to increased pain and eventually form and maintain psychological disorders (55). Acceptance is the opposite of experiential avoidance, which can alleviate and regulate human suffering and reduce the negative effects of psychological disorders (18). Therefore, acceptance can alleviate the negative feelings of depression, including those of pain and hopelessness, and thus reduce the effect of depression on SI.

Furthermore, according to the Experiential Avoidance Model of deliberate self-harm, individuals can strengthen and maintain self-harm behaviors by escaping or avoiding unwanted emotional experiences such as depression, while adopting acceptance to face adverse emotional reactions such as depression can alleviate self-injurious behaviors (57). Self-harming behavior is highly correlated with SI, and adolescents who intentionally self-harm have significant suicide risk (76). In summary, experiential avoidance can enhance the effect of depression on SI, while acceptance can alleviate the effect of depression on SI.

## Limitations

The current study expanded our understanding of the moderated mediation between family functioning and SI of college students, especially revealed the moderating effect of acceptance on depression and SI, and verified the role of acceptance commitment therapy theory in suicide prevention. Despite the advantages mentioned above, there are some limitations, there are nonetheless some limitations. First, the cross-sectional study failed to take into account the variation of time series of variables. The moderated mediation mechanism between family functioning and SI should be studied by means of a longitudinal study in the future. Second, this study used self-reported data via questionnaires which can lead to errors due to the effect of social expectations. Future studies should consider the combination of self-reported questionnaire and other evaluation questionnaire formats to improve the validity of the study. Third, as mentioned in the current study, demoralization, as a more inclusive concept of mental pain, is attracting more and more attention. Future studies should pay attention to the relationship between demoralization and acceptance, especially in the generation of suicidal ideation. Fourth, the formation of SI is also influenced by biological factors, which should be taken into account in future studies. Finally, the sample in this study comprised students from only one university in one province in China, and as such, the results may not be generalized to college students from other cultural backgrounds. Therefore, sample representativeness should be improved in the future, especially incorporating college students from different cultural backgrounds.

## Implications for practice

Despite the above-mentioned limitations, this study nonetheless has implications for preventive interventions. First, depression is the mediating variable between family functioning and SI. Alleviating the depressive emotion of college students could help prevent college students from committing suicide. Ways to achieve this could include carrying out a “gatekeeper” program in Chinese colleges and universities, strengthening the psychological survey of college and university students, paying attention to the depression of college students, and providing appropriate help to depressed college students, all of which could be conducive to reducing the depression of college students. Second, acceptance was shown to help mitigate the effects of depression on SI. Therefore, college students should avoid excessive use of experiential avoidance strategies, and instead adopt more effective emotion regulation strategies and enhance their acceptance of their current feelings and experiences. For example, college students could be taught to make use mindfulness, distress tolerance, and radical acceptance skills through mental health education courses.

## Conclusion

This study reveals the role of depression and acceptance in the relationship between family functioning and SI in Chinese college students. The results showed that depression was the mediating variable between family functioning and SI. Acceptance plays a moderating role in the relationship between depression and SI in college students, and can effectively alleviate the influence of depression on SI. The results of this study provide guidance for mental health workers in colleges and universities to prevent and intervene in incidences of college students suicides.

## Data availability statement

The raw data supporting the conclusions of this article will be made available by the authors, without undue reservation.

## Ethics statement

The studies involving human participants were reviewed and approved by the Ethics Committee of Guizhou Medical University. The patients/participants provided their written informed consent to participate in this study.

## Author contributions

BP and HY conceived and designed the research. BP performed the statistical analysis and drafted the manuscript. NH completed the literature review and interpretation of the results. LG participated in the study design and interpretation analysis. CC collected the data. ZC makes a significant contribution to the conception of the research and provides important guidance for the revision of the article. All authors contributed, read, and approved the final manuscript.

## Funding

This work was funded by the Major Research Base of Humanities and Social Sciences (23RWJD270), Education Department of Guizhou Province.

## Conflict of interest

The authors declare that the research was conducted in the absence of any commercial or financial relationships that could be construed as a potential conflict of interest.

## Publisher’s note

All claims expressed in this article are solely those of the authors and do not necessarily represent those of their affiliated organizations, or those of the publisher, the editors and the reviewers. Any product that may be evaluated in this article, or claim that may be made by its manufacturer, is not guaranteed or endorsed by the publisher.
